# Discrepancy of Growth Toxicity of Polystyrene Nanoplastics on Soybean (*Glycine max*) and Mung Bean (*Vigna radiata*)

**DOI:** 10.3390/toxics12020155

**Published:** 2024-02-17

**Authors:** Dan Su, Wangwang Li, Zhaowei Zhang, Hui Cai, Le Zhang, Yuanlong Sun, Xiaoning Liu, Zhiquan Tian

**Affiliations:** 1 College of Chemistry and Molecular Sciences, Wuhan University, Wuhan 430072, China; 2 School of Ecology and Environment, Tibet University, Lhasa 850000, China; 3 State Key Laboratory of New Textile Materials and Advanced Processing Technologies, School of Bioengineering and Health, Wuhan Textile University, Wuhan 430200, China; 4 State Key Laboratory of Water Resources Engineering and Management, Wuhan University, Wuhan 430072, China

**Keywords:** soybean, mung bean, polystyrene nanoplastics, phytotoxicity, oxidative stress

## Abstract

Nanoplastics, as a hot topic of novel contaminants, lack extensive concern in higher plants; especially the potential impact and mechanism of nanoplastics on legume crops remains elusive. In this study, the toxicity of polystyrene nanoplastics (PS-NPs, 200 nm) with diverse doses (control, 10, 50, 100, 200, 500 mg/L) to soybean and mung bean plants grown hydroponically for 7 d was investigated at both the macroscopic and molecular levels. The results demonstrated that the root length of both plants was markedly suppressed to varying degrees. Similarly, mineral elements (Fe, Zn) were notably decreased in soybean roots, consistent with Cu alteration in mung bean. Moreover, PS-NPs considerably elevated malondialdehyde (MDA) levels only in soybean roots. Enzyme activity data indicated mung bean exhibited significant damage only at higher doses of PS-NPs stress than soybean, implying mung bean is more resilient. Transcriptome analysis showed that PS-NPs stimulated the expression of genes associated with the antioxidant system in plant roots. Furthermore, starch and sucrose metabolism might play a key role in coping with PS-NPs to enhance soybean resistance, but the MAPK pathway was enriched in mung bean. Our findings provide valuable perspectives for an in-depth understanding of the performance of plants growing in waters contaminated by nanoplastics.

## 1. Introduction

Plastic products are widely produced and demanded due to their light weight, low cost, and durability, thereby increasing the likelihood of posing a threat to the environment [1]. Plastic particles less than 5 mm in diameter are commonly described as microplastics (MPs) and classified into primary and secondary types [2]. The primary types refer to micron-sized plastics that are artificially manufactured, including common cleaning products like exfoliants and industrial abrasives, whereas physical abrasion, chemical reactions, and biodegradation with larger fragments in the surroundings such as tire wear or the degradation of disposable coffee lids form the secondary types [3,4]. To date, the potential harms of microplastics have emerged as a prominent focus owing to their small size, expansive surface area, and enhanced ability to absorb pollutants [5]. Furthermore, various types of polymers in microplastics are detected in diverse areas, especially marine ecosystems, terrestrial systems, the atmosphere, and even human blood, which suggests a potential hazard to both the environment and human health [6,7,8,9].

The ubiquity of microplastics in aquatic habitats and terrestrial systems has drawn considerable attention. Especially for aquatic areas that play an irreplaceable role, the presence of MPs may cause water pollution, rendering the water unusable or unsuitable for organisms [10]. According to the report, the biomass and growth rates of *Lumbricus terrestris* were significantly degraded by exposure to litter containing 60% *w*/*w* polyethylene microplastics [11]. MPs can also have an adverse impact on cytogenetics [12], stress responses [13], reproductive deficiencies [14], and other biomarker responses. Furthermore, there is direct evidence that an estimated roughly 6.5 × 10^8^ MPs from wastewater treatment plants (WWTPs) are released into Xiamen Bay each day, revealing that the WWTPs may substantially elevate the number of MPs in the water environment [15]. It’s worth mentioning that polystyrene (PS) microplastics were determined at a high frequency in treated water-supplying water treatment plants, and the Toxicology Research Survey 2019 indicated that 50% of the research conducted is primarily focused on PS microplastics [16,17]. Consequently, broadly dispersed and sharply focused PS microplastics were chosen as the test material for this trial.

Hydroponics is a common approach for testing the toxicity of microplastics on organisms. Initially, Bosker et al. found the physical blockage of the seed capsule pores of *Lepidium sativum* by microplastics for the first time [18]. Moreover, previous reports have indicated the impact of microplastics on plants’ phenotypic indexes. For instance, wheat seedlings under polystyrene stress reduced the shoot to root biomass ratio [19]. There is also direct evidence of the absorption and transportation of microplastics in plant tissues. For example, 200 nm PS-NPs were reported in the roots and translocated the vascular bundles of petioles through a xylem-based upward translocation pathway, posing a significant influence on the antioxidant, osmoregulation, and photosynthetic systems of strawberry seedlings [20]. Furthermore, polystyrene triggered oxidative stress in *Oryza sativa* under hydroponic conditions and affected seed growth by modulating amino acid metabolism [21]. Scientific research personnel generally choose seeds of common food crops (rice, wheat) as research targets for microplastic toxicology experiments. However, a handful of legume crops such as fava beans have been observed to undergo significant oxidative damage and genotoxicity when exposed to pharmaceutical container leachate [22]. In addition, the current report on lentil seeds of the internal activity at the initial stages was restrained by polyethylene microplastic stress [23]. So far, a tiny minority of studies have provided a molecular explanation for the mechanisms of soybean response to PS-NPs and perfluorooctane sulfonate (PFOS) co-stress [24]. According to the previous research, more noteworthy is that the discrepancy in the response and mechanisms among various legume crops to PS-NPs stress still constitutes a scientific gap.

Soybean and mung bean sprouts are widely consumed vegetables that are rich in protein and amino acids, with a short reproductive period, which plays a crucial role in diversifying people’s dietary patterns [25]. During hydroponics, once sprouts are exposed to pollution factors, including the selection of the water source or the transportation of water supply pipes, the quality of the sprouts may be adversely affected, even absorbing pollutants. Hence, the present study was conducted to investigate the impacts of diverse doses (control, 10, 50, 100, 200, and 500 mg/L) of PS-NPs on soybean and mung bean grown hydroponically at the macroscopic and molecular levels, including seed growth parameters, physiological indicators, SEM observations, mineral element levels, and transcriptome analysis.

## 2. Materials and Methods

### 2.1. Preparation of Nanoplastics and Seeds

Soybean (*Glycine max*) and mung bean (*Vigna radiata*) seeds were obtained from a farmer’s market in Wuhan, China. The PS emulsion was purchased from Shanghai Huge Biotechnology Co. Ltd. (Shanghai, China). The PS particles of appropriate doses were dispersed in distilled water and sonicated for 10 min for subsequent characterization. The morphology of PS particles was characterized with a transmission electron microscope (JEM-2100 Plus, JEOL, Tokyo, Japan). The hydrodynamic diameter and zeta potential of PS suspensions were determined using a dynamic light scattering instrument (Nano ZS, Malvern, UK). The structure and composition of particles were analyzed via a Fourier transform infrared spectrometer (FTIR5700, Thermo Fisher Scientific, Waltham, MA, USA).

The PS emulsion was diluted with doses of deionized water to gain six dilutions (0, 10, 50, 100, 200, and 500 mg/L) as culture suspensions. Smooth, uniformly sized Soybean (*Glycine max*) and mung bean (*Vigna radiata*) seeds were meticulously chosen and sterilized by standing in 3% hydrogen peroxide (H_2_O_2_) for 20 min and then rinsed multiple times with ultrapure water to eliminate the residue. The sterilized seeds were germinated for 8 h in a dark environment at 25 °C for later cultivation.

### 2.2. Hydroponic Design

A total of 12 treatments were conducted in this trial, with at least three duplicates for each treatment. The PS-induced toxicity test was slightly modified according to the protocol of Wang et al. [26]. The pre-sterilized wet double-layer filter paper and 10 seeds were sequentially transferred into Petri dishes (9 cm in diameter). Subsequently, the six pre-homogenized 10 mL suspension of PS were separately added to each treatment. After that, the cultures were arranged in a night incubator with 25 °C and humidity of 70% for 7 d, then 5 mL of the PS suspension was added each day to compensate for water evaporation.

### 2.3. Measurement Parameters

#### 2.3.1. Growth and Biochemical Indexes of Sprouts

After 7 d, the sprouts of soybean and mung bean were washed with ultrapure water to remove any residue, respectively. The root length of sprouts was measured using a millimeter ruler, and the weight was subsequently determined using an analytical balance with a precision of one part in ten thousand. The sprouts were then transferred to a refrigerator at a temperature of −80 °C for 8 h, followed by a freeze-drying process for 48 h. Ultimately, the dried weights of sprouts were documented.

Given the short incubation period of sprouts, solely the root system was investigated in our study. The root tissues (0.1 g) were quick-frozen in liquid nitrogen for enzyme activity experiments. The kits for assessing enzyme activity were purchased from Beijing Solar Bioscience & Technology Co., Ltd. (Beijing, China). The activity of sprouts superoxide dismutase (SOD) was determined using the nitrogen blue tetrazolium (NBT) reduction assay, while the activity of peroxidase (POD) was assessed through the guaiacol colorimetric assay. Catalase (CAT) plays a crucial role in scavenging H_2_O_2_, which content by monitoring the alteration in absorption at 240 nm. The 2-thiobarbituric acid reaction is used to quantify Malondialdehyde (MDA) levels.

#### 2.3.2. Elemental Content of Sprouts

Approximately 0.15 g of dried root tissue powder was transferred into a beaker containing 4 mL of nitric acid overnight. The mixture was heated on a stirring hotplate at 200 °C for 3 h, and 2 mL of H_2_O_2_ was gradually added during the digestion process, with the solution evaporating to 1 mL using residual heat. The solution was deliquated to 10 mL with Milli Q water and then filtered through 0.22 μm filters to quantify the element content of Magnesium (Mg), Iron (Fe), Copper (Cu), and Zinc (Zn) via inductively Coupled Plasma-Atomic Emission Spectrometry (ICP-OES) (Agilent 5110, Agilent, Santa Clara, CA, USA).

#### 2.3.3. SEM Observations

The harvest roots of soybean and mung bean growing in 50 mg/L PS-NPs solution for 7d were sliced into 0.1–0.2 mm pieces and then immersed in 2.5% glutaraldehyde fixative at 4 °C [27]. Subsequently, post-fixed samples were dehydrated using different doses of ethanol gradients. The treated root tissues were frozen in liquid nitrogen for 30 min and vacuum freeze-dried for 48 h. Dried samples were coated with a layer of gold for 60 s using a magnetron sputter coater (SC7620, Quorum, Edinburgh, UK). Finally, the tissue cross-sections were imaged under a scanning electron microscope (GeminiSEM 300, Zeiss, Oberkochen, Germany) at various magnifications, with triplicate motion.

### 2.4. Transcriptome Analysis

The roots of soybean and mung bean were exposed to different doses of PS-NPs (100, 500 mg/L) treatment groups (named G100, G500, V100, and V500), and the control check (named GCK, VCK) were sequenced, respectively. Fresh root tissues were rinsed with RNase-free water and transferred into 5 mL RNase-Free EP tubes. The tubes were snap-frozen in liquid nitrogen for 30 min and immediately stored in a −80 °C refrigerator. Total RNA extraction, mRNA enrichment using Oligo (dT), mRNA fragmentation, cDNA reverse synthesis, adapter ligation, PCR amplification, library detection, and cyclization were performed by BGI Shenzhen Co., Ltd. (Wuhan, China). The DNBSEQ platform (DNB, Shenzhen, China) was utilized for sequencing, and the data were analyzed on the Dr. Tom platform (https://report.bgi.com).

### 2.5. Statistical Analysis

All experiments included at least three duplicates. Shapiro–Wilk test and Levene’s test were screened for normality of data in this study. The results were expressed as means ± standard deviation (Means ± SD). Statistical analyses were completed with SPSS 17 software (IBM, Armonk, NY, USA) and illustrations were produced using Origin 2021. Tukey’s post hoc test was employed in a one-way analysis of variance (ANOVA) to assess group differences, with a significance level of *p* < 0.05.

## 3. Results and Discussion

### 3.1. Characterization of NPs

The particles exhibit a smooth spherical morphology and are uniform in size (Figure S1A), with an average diameter of 200.7 ± 6.1 nm (Figure S1B). The polymers were verified to be PS-NPs without other surface functional groups (Figure S1C) [28]. The size and zeta potential of the PS suspension were analyzed in Figure S1D. Notably, the hydrated particle size of the PS suspension resembled the microsphere of the TEM value, indicating that the PS-NPs are uniformly dispersed in ultrapure water. In addition, according to the DLVO theory, the zeta potential of the PS suspension ranged from −30 to −60 mV, confirming the stability of the dispersed system [21,29].

### 3.2. Alterations in the Phenotypic Parameters of Sprouts

**Figure 1:**
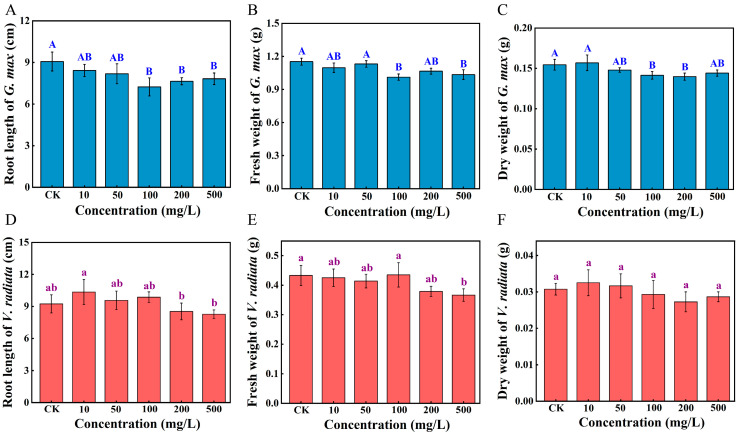
Effects of PS-NPs on the root length (**A**,**D**), fresh weight (**B**,**E**), and dry weight (**C**,**F**) of soybean and mung bean on the 7th day. Data represent means ± SD. Distinct letters denote statistically significant differences between diverse concentrations (*p* < 0.05). Note: uppercase letters and lowercase letters correspond to soybean and mung bean, respectively.

After 7 d of exposure, compared with GCK, the root length of the soybean sprouts was significantly suppressed by 20.2% at 100 mg/L of PS-NPs and attenuated inhibition at the highest concentration (Figure 1A, *p* < 0.01), whereas markedly reduced by 10.5% at 500 mg/L in mung bean sprouts (*p* > 0.05) and mildly promoted at low concentrations (Figure 1D, *p >* 0.05). There was no significant change in the length of sprout roots when soybean seeds were exposed to PS-NPs with concentrations below 100 mg/L, compared to the unexposed groups (*p* > 0.05). Evidently, there is a noticeable diminution in the fresh and dry weight of soybean upon exposure to 100 mg/L of PS-NPs by 11.5% and 9.4%, respectively (Figure 1B,C, *p* < 0.05). As shown in Figure 1E, the fresh weight of mung bean exposed to 500 mg/L of PS-NPs was 15.4% lower than VCK (*p* < 0.05). Additionally, there were no notable changes in the dry weight of mung bean (Figure 1F, *p >* 0.05). Plant biomass is one of the common indicators for the assessment of the extent of plant response to stress [12]. In line with the soybean findings, the shoot length of lettuce exposed to a medium (10^3^ particles/mL) dose of PS had a maximum inhibition of 36% compared to the control [30]. This phenomenon is generally explained by the possibility that PS-NPs may attain saturation at a certain concentration, leading to the no-longer increasing toxic effects [31]. Additionally, PS-NPs at a concentration of 10 mg/L slightly promoted the growth of mung bean roots, whereas high concentrations of PS-NPs inhibited the growth of mung bean roots (*p* > 0.05). These findings are consistent with those of Lian and Zhang et al. [19,29]. However, the mechanism by which low concentrations of PS-NPs promoted plant root growth was unclear, and a possible reason for this phenomenon is that PS-NPs stimulate the secretion of certain hormones (e.g., growth hormone) in the mung bean roots, which may help mung beans adapt to environmental changes and maintain their physiological functions. Nevertheless, high concentrations of PS-NPs may block the pores of the mung bean seed capsule, thereby preventing water and nutrient uptake by the seeds and further inhibiting the root of mung bean sprouts [18]. Furthermore, soybeans exhibited a more sensitive response to the dose of PS-NPs than mung beans. Previous studies have confirmed that Italian lettuce under microplastic stress was the most responsive to seed germination of four crops (Italian lettuce, radish, wheat, and maize) [32]. One possible explanation is that the root surfaces of different plants adsorb nanoplastics at diverse doses, resulting in discrepancies in their uptake or in the utilization of essential nutrients [18,33]. Moreover, soybean and mung bean belong to diverse plant families with high genetic variety and may respond differently to the exoteric environment [34]. There were varying degrees of variations in the growth indexes of soybean and mung bean among all the PS treated groups, implying that PS-NPs had a negative impact on sprouts.

### 3.3. Alterations in the Oxidative Stress Response of Sprouts

**Figure 2:**
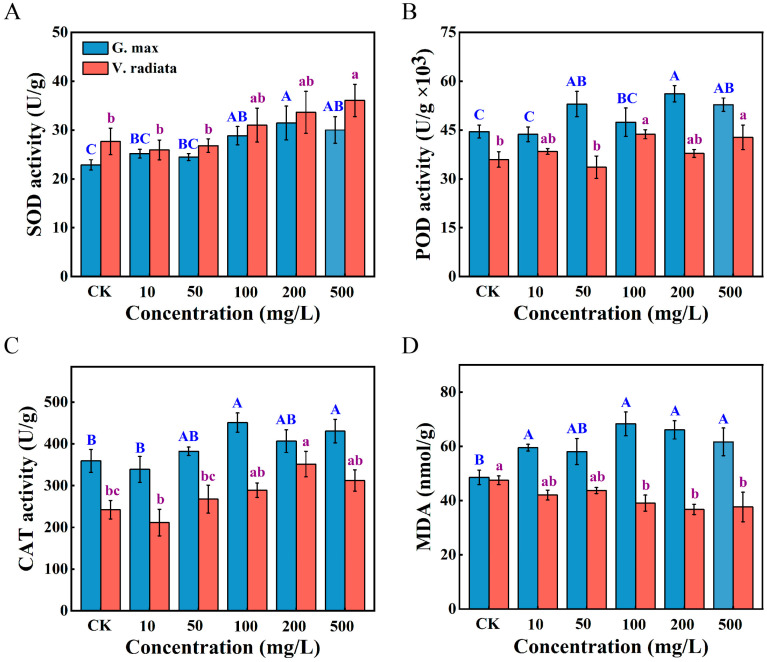
SOD (**A**), POD (**B**), and CAT (**C**) activities, and MDA (**D**) content of soybean and mung bean roots as a result of different PS-NPs exposure levels. Data represent means ± SD. Distinct letters denote statistically significant differences between diverse concentrations (*p* < 0.05). Note: uppercase letters and lowercase letters correspond to soybean and mung bean, respectively.

Exposure to PS-NPs elicited distinct stress responses in the roots of soybean and mung bean sprouts (Figure 2). A significant enhancement of SOD and CAT activities was recorded in soybean roots at 100 mg/L of PS-NPs (*p* < 0.05, *p* < 0.01, respectively), and showed a positive correlation in mung bean under PS-NPs concentrations from 50 to 500 mg/L as a whole (Figure 2A,C). With a similar pattern to that in previous reports [35], no marked changes in SOD, CAT, and POD levels were observed in soybean and mung bean under exposure to 10 mg/L doses of PS-NPs (*p* > 0.05). The POD activity of soybean when exposed to 50 mg/L of PS-NPs was dramatically activated (*p* < 0.05), while a notable increase was only observed at 100 and 500 mg/L compared to the control in mung bean (Figure 2B, *p* < 0.05). The MDA content is presented in Figure 2D. Notably, the MDA content of soybean roots exhibited a striking increase in all treatments with PS-NPs except for 50 mg/L, and the highest increment at the dose of 100 mg/L, compared to the unexposed groups (*p* < 0.01). On the contrary, the MDA content generally presented a noticeable decreasing inclination with the continuous increase of PS-NPs doses in mung bean (*p* < 0.05).

Plants produce an excessive quantity of reactive oxygen species (ROS) triggered by both biotic and abiotic stressors and mitigate the effects of external perturbations on their biological processes by regulating the antioxidant enzyme system [36]. The SOD and CAT, existing in animals, plants, and microorganisms, are the plant’s first line of self-defense against stress [37]. The changes in SOD content showed that soybean and mung bean formed differences in response to PS-NPs stress, with available support [38]. Additionally, the activation of POD activity in mung bean requires exposure to higher doses of contaminants than soybean, suggesting that mung bean may have a stronger tolerance. The increase in CAT level may be attributed to the fact that mung bean resistance to high doses of PS-NPs increases SOD content, which can result in the mass production of H_2_O_2_ [23]. This may be the main detoxification mechanism for mung bean. Lipid peroxidation, which may generate small molecules such as MDA, is a significant indicator evaluating cell membrane damage when encountering unfavorable environments in plants [12,39]. The rise in the MDA content of soybean may be related to the high concentration of ROS induced by PS-NPs, which attacks the unsaturated double bonds of unsaturated fatty acids on the phospholipid molecules, leading to membrane lipid peroxidation in the cell membrane [40,41]. Furthermore, a similar result by Zhang et al. [20], the reduction in MDA content may be due to the scavenging capacity of the antioxidant system lower than excessive ROS produced [42]. In general, the discrepancy in MDA content was understood as the antioxidant systems playing an important role in adapting heavy metal stress in *Kandelia candel* and *Bruguiera gymnorrhiza* species by Zhang et al. [38]. Currently, widespread evidence indicates that the surface charge, size, and concentration of microplastics are intimately linked to the effects of growth processes in diverse plant species [5,30]. The biochemical responses of plants are influenced by the doses of PS-NPs and legume species, as supported by our findings.

### 3.4. Alterations in Mineral Elements of Sprouts

**Figure 3:**
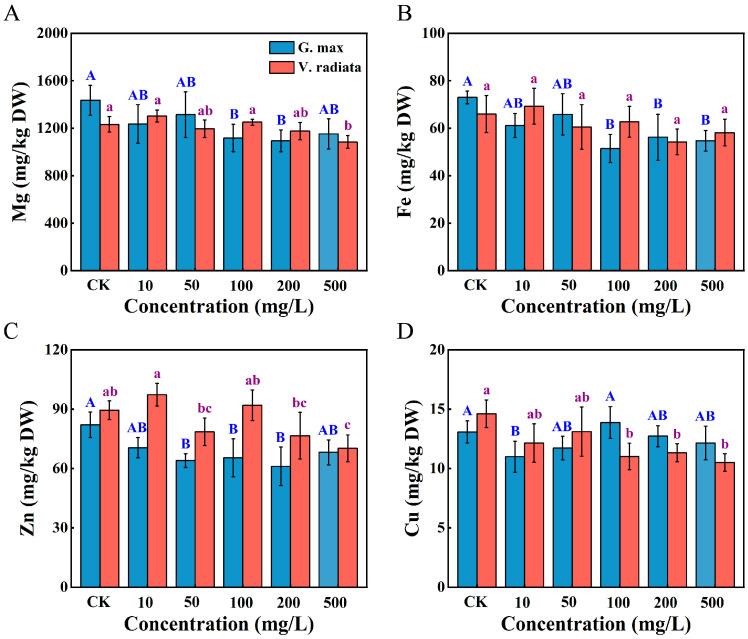
Effect of mineral elements of soybean and mung bean roots exposed to PS-NPs, the levels of Mg (**A**), Fe (**B**), Zn (**C**), and Cu (**D**). Data represent mean ± SD. Distinct letters denote statistically significant differences between diverse concentrations (*p* < 0.05). Note: uppercase letters and lowercase letters correspond to soybean, and mung bean, respectively.

Mineral elements (Mg, Zn, Fe, and Cu), which act as enzyme activators or electron carriers, are essential for plant growth, and external disturbances can affect the uptake and translocation of these nutrients in plants [43]. The effect of PS-NPs on mineral elements was assayed in the roots of soybean and mung bean sprouts (Figure 3). Compared to VCK, the Mg, Fe, and Zn contents of soybean exposed to 10 mg/L declined, whereas increment in mung bean (*p* > 0.05). Regarding macronutrients, remarkable reductions of Mg content in soybean were observed at 100 and 200 mg/L of PS-NPs (*p* < 0.05), while a significant decrease only at 500 mg/L in mung bean, compared to those of control (Figure 3A, *p* < 0.05). One of the potential mechanisms may be that microplastics blockage seed pores, preventing water and nutrient uptake [19]. The micronutrient Fe content of soybean exposure ranging from 100 to 500 mg/L of PS-NPs was significantly decreased, consistent with the Zn variation (Figure 3B, *p* < 0.05). The alterations in Zn and Fe levels may be due to soybean coping with PS-induced stress. Moreover, the reduction of Fe and Zn content negatively affects the enzymatic antioxidant capacity of lettuce to the study of Lian et al. [44]. Hence, the alterations in enzyme activities observed in soybean roots may be attributed to the combined impact of variations in mineral elements and PS-NPs stress in this experiment. Additionally, saliency depressing in Zn content of soybean was observed at 50 mg/L (*p* < 0.05), nevertheless marked degraded only at 500 mg/L in mung bean with respect to the control (Figure 3C, *p <* 0.05). Different plants exhibit specific responses to stress, which may explain the varying effects of PS-NPs in the Zn uptake of soybean and mung bean roots. However, the Cu content was markedly lower than GCK in soybean under only 10 mg/L of PS-NPs (Figure 3D, *p* < 0.05), while displaying a negative correlation with the doses of PS-NPs in mung bean. Cu element has been demonstrated a key role in enhancing plant resistance to pathogens through lignification, callus formation, and ROS induction [45]. The decrease in Cu levels of soybean and mung bean by PS-NPs stress, which may have been detrimental to plants’ disease resistance. Moreover, several aquaporins and major ion transporters (e.g., ion transmembrane transport or metal ion transport) are accountable for plant nutrient uptake [46,47]. Altogether, the roots of soybean and mung bean exposed to PS-NPs exhibited varying changes in the content of the four mineral elements, suggesting PS-NPs may selectively inhibit the uptake of essential nutrients of sprouts.

### 3.5. Scanning Electron Microscope Observation

**Figure 4:**
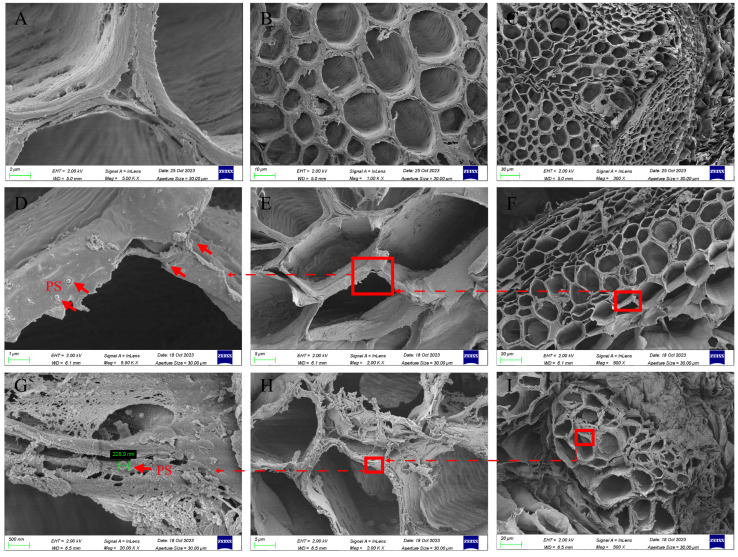
Scanning electron microscopy (SEM) images of roots in soybean (**D**–**F**) and mung bean (**G**–**I**) exposed to 50 mg L^−1^ polystyrene (PS) nanoplastics for 7 d and the control of soybean (**A**–**C**). Note: (**A**,**D**,**G**) are partial enlargements of (**B**,**E**,**H**), respectively; (**B**,**E**,**H**) are partial enlargements of (**C**,**F**,**I**), respectively.

After 7 d of exposure to 50 mg/L PS-NPs, we performed SEM observations of the roots of soybean and mung bean. Sections from the control of soybean roots showed no detectable PS-NPs (Figure 4A–C). For the PS-NPs treatment group, the sphere particles were agglomerated in the cortex of the soybean (Figure 4D–F). Furthermore, soybean roots of the vascular bundle were found in the presence of sphere particles (Figure S2A–C), proving that PS-NPs in size of 200 nm can penetrate the root epidermis of the soybean and enter the vascular bundle, consistent with Zhang et al. [20]. In theory, only particles ranging from 5 to 20 nm in size can penetrate the cell wall [48]. However, direct evidence showed that PS with different particle sizes (such as 0.1, 0.3, and 1 μm) can be internalized by plant roots and even transported to the above-ground parts [21,45]. In addition, our evidence showed that PS-NPs can penetrate the cortex of sprouts. However, quantifying the concentration of PS-NPs in sprouts remains challenging, including the relationship between the initial PS-NPs concentration and the number of PS particles that penetrate the plant cortex, as well as establishing standard methods for quantification. So far, researchers and scholars have proposed possible mechanisms for the entry of microplastics into plants. For instance, Li et al. indicated that microplastics penetrate the stele of wheat and lettuce species through the crack-entry mode at sites on the lateral root, which allowed PS-NPs to penetrate the root cortex and be transported from the root to the shoots by transpiration pulling forces [49]. The pathway of PS-NPs entering plants may be limited to specific plant research objects, and due to significant physiological and structural differences between plant species, it may not be possible to determine whether other species also penetrate plant cortical tissues in this way. In the present study, the detection of PS-NPs occurred in the vascular bundles of soybean roots, suggesting that particles may be translocated through the apoplastic pathway [24]. However, no evidence of the upward transport of PS-NPs was found in soybean. Moreover, sphere particles were not detected in mung bean roots without PS-NPs treatment (Figure S2D–F). Similar to soybean, a minute quantity of spheres was captured in the vascular system of mung bean roots (Figure 2G–I), indicating that PS-NPs also penetrate the epidermis of mung bean. Nanoparticles give strong adhesion properties and can be efficiently ‘captured’ by the polysaccharide mucus secreted by the plant root system, which could potentially elucidate the reason behind the obstruction of the majority of sphere particles outside the seed epidermis [50]. Additionally, it was also reported that a minor deformation of nanoparticles was observed in the root of cucumber [51], but no deformation of particles was noticed in the roots of the sprouts in the present study. Our results imply that 200 nm PS-NPs can overcome biological barriers and enter the roots of soybean and mung bean.

### 3.6. Transcriptome Analysis

#### 3.6.1. Identification of Differentially Expressed Genes

**Figure 5:**
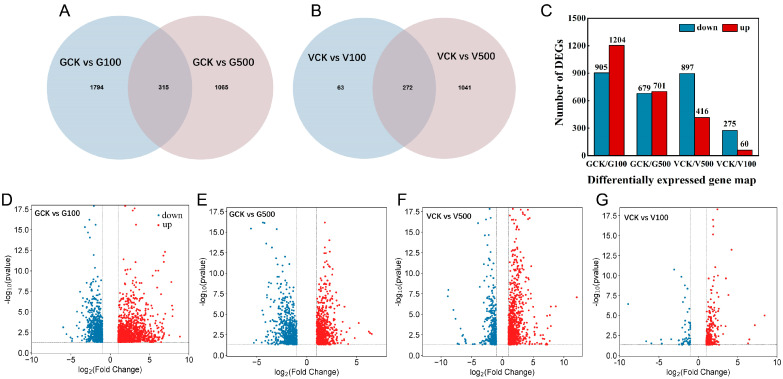
The distribution of DEGs in soybean (**A**,**D**,**E**) and mung bean (**B**,**F**,**G**) roots exposed to PS-NPs for 7 d. Venn diagram/bar plot (**A**–**C**), and volcanic maps (**D**–**G**). Red plots depict upregulated genes; blue plots depict downregulated genes.

RNA sequencing (RNA-seq) of the transcriptome has emerged as a vital means of exploring the physiological and molecular mechanisms of the plant stress response to microplastics [52]. In this experiment, we performed differential gene analyses (DEGs, |fold change| ≥ 2) in soybean and mung bean roots (Figure 5). The Venn diagrams show the co-expressed and exclusively expressed genes in circles within a comparison group, whereas the number in the overlapping regions of the circles represents the number of common differential genes among two corresponding comparison combinations (Figure 5A,B). Compared to the control, a total of 2109 genes (GCK vs. G100, 1204 upregulated, and 905 downregulated) and 1380 genes (GCK vs. G500, 701 upregulated, and 679 downregulated) were detected in the soybean roots, respectively, implying that the GCK vs. G100 group stimulated more PS-NPs and changed more genes than the GCK vs. G500 group. Moreover, the VCK vs. V100 groups revealed 1313 expressed genes (416 upregulated, 897 downregulated), and a total of 335 genes were expressed in the VCK vs. V500 groups (60 upregulated, 275 downregulated). Subsequently, the DEG distributions and discrepancy multipliers of the four groups were investigated using volcano plots (Figure 5D–G).

#### 3.6.2. Enrichment Analysis of Differential Genes

**Figure 6:**
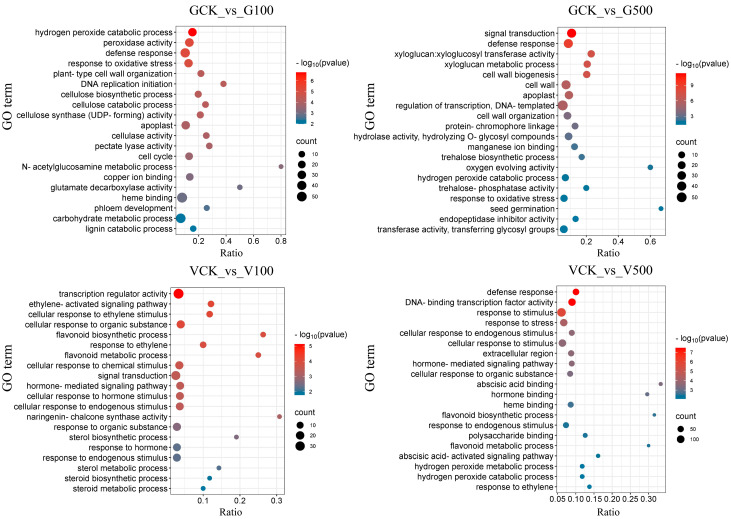
Bubble diagram of GO terms in the roots of soybean and mung bean. The y-axis displays the term name, and the x-axis displays the gene ratio (rich factor) for each term. The size of the point shows the number of genes in the GO term.

**Figure 7:**
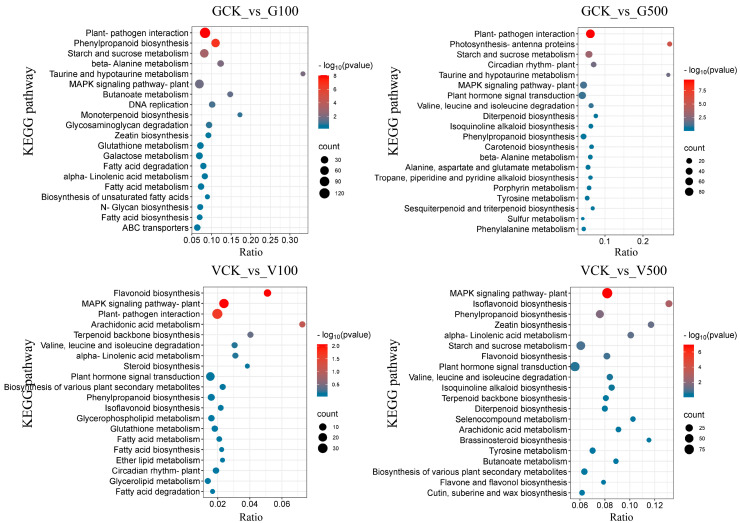
Bubble diagram of the KEGG term in the roots of soybean and mung bean. The y-axis displays the term name and the x-axis displays the gene ratio (rich factor) for each pathway. The size of the point shows the number of genes in the KEGG pathway.

Gene ontology (GO) enrichment (Figure 6) and the Kyoto Encyclopedia of Genes and Genomes (KEGG) pathway (Figure 7) were analyzed on differential genes in the roots of soybean and mung bean. The gene expression was different in soybean and mung bean exposed to diverse doses of PS-NPs. The findings showed that the GCK vs. G100 group and GCK vs. G500 group dominantly showed common enrichment terms, including the response to oxidative stress and the defense response. Highly similar results were reported by Zhang et al., showing that oxidative stress may be a coping mechanism for soybean to resist negative external stimuli [53,54]. Specifically, the hydrogen peroxide catabolic process was enriched in the GCK vs. G100 group. Elevated levels of hydrogen peroxide, as an oxidative stress product, may cause cell damage or even death [55]. Consistent with our previous results, CAT activity, as a hydrogen peroxide scavenging enzyme, had a remarkable increase, indicating that PS-NPs activate the antioxidant system in soybean (Figure 2). In the PS-NP treatment group, flavonoid processes (biosynthetic and metabolic) were enriched to varying degrees, which may be attributed to differences in the dose of PS-NPs. Additionally, it was found that the VCK vs. V100 group exhibited a significant enrichment of the ethylene signaling pathways. These pathways encompassed the ethylene-activated signaling pathway, response to ethylene, and cellular response to ethylene stimulus. Ethylene is a gaseous plant hormone that influences growth processes such as germination, nodulation, programmed cell death, stress response, and pathogen attack [56]. Mung bean may respond to the interference of PS-NPs by regulating plant hormones. More importantly, the cellular response (endogenous stimulus and organic substance) terms were enriched in the VCK vs. V500 group, suggesting that plant cells may maintain a dynamic equilibrium by responding hormonally, or maintain nutrients to ensure the normal execution of cellular physiological functions by stress [57]. Similar to soybean, the defense response was enriched in the VCK vs. V500 group. Overall, the response of soybean to external environmental intervention may primarily involve the regulation of the antioxidant system and oxidative stress response, whereas it may mainly involve the regulation of flavonoids, cellular response, and ethylene signaling pathways in mung bean.

The results revealed that plant–pathogen interaction and starch and sucrose metabolism were the co-enrichment pathways in two comparison groups of soybeans with PS-NPs treatments. The effects of the plant–pathogen interaction contributed to enhancing the plant’s ability to tolerate external disturbances and stress [58]. Furthermore, previous studies have shown that the starch and sucrose metabolism pathway varied with the salt treatment process in Aquilegia, which can affect the distribution of sugar-energy sources [59]. The alteration of mineral elements in soybean roots may be attributed to the obstruction of energy conversion in seeds caused by PS-NPs (Figure 3). Furthermore, the phenylpropanoid biosynthesis and beta-alanine metabolism pathways promoting plant growth and enhancing resistance against pathogenic microorganisms and abiotic stresses were activated in the GCK vs. G100 group [53]. Additionally, GCK compared with G500 simultaneously affected the circadian rhythm of plant and photosynthesis-antenna proteins pathway. These results imply that PS-NPs enhanced the process of photosynthesis and the production of certain plant secondary metabolites in plants. In the comparison groups of mung bean, we observed the co-enrichment of the MAPK signaling pathway. This pathway is crucial in regulating various physiological processes, such as cellular growth, adapting to environmental stress, and responding to inflammation, which may be initiated by pathogenic infections [40]. Moreover, the isoflavone biosynthesis pathways were enriched in the VCK and V500 group, suggesting that mung bean may regulate isoflavone biosynthesis as a means of resisting stress. The isoflavone biosynthesis pathway is a complex biochemical reaction that enhances the plant’s disease resistance and maintains normal physiological functions [60]. In the VCK vs. V100 group, the pathways related to flavonoid biosynthesis and plant–pathogen interaction exhibited enrichment. These pathways facilitate the removal of reactive oxygen species through non-enzymatic antioxidants, ensuring the normal structure and function of cells [61]. The findings indicate that starch and sucrose metabolism and the plant–pathogen interactions pathway might play a key factor in coping with PS-NPs to enhance soybean resistance, but mung bean were predominantly triggered in the MAPK and flavonoid biosynthesis pathway. In general, the above results indicated that soybeans were more susceptible to PS-NPs stress than mung beans, and the same types of plants exhibited differential responses to varying doses of PS-NPs stress.

## 4. Conclusions

The current study aimed to assess the phytotoxicity of PS-NPs on the growth of soybean and mung bean, in combination with macro-parameter and transcriptome analysis. The findings pointed out that PS-NPs could significantly hinder root length, biomass, and the mineral element (Mg, Fe, Cu, and Zn) parameters of soybean and mung bean to varying degrees. Moreover, exposure to PS-NPs in soybean produces more ROS and induces higher oxidative damage than in mung bean. Transcriptome analysis demonstrated that soybean activated the starch and sucrose metabolism pathways to combat PS-NPs stress, whereas mung bean predominately activated the MAPK signaling pathways to regulate the damage caused by PS-NPs. Overall, the findings in this study highlighted that soybean were more susceptible to PS-NPs stress than mung bean, providing valuable perspectives for an in-depth understanding of the performance of crops growing in waters contaminated by nanoplastics.

## Data Availability

Data are contained within the article.

## Supplementary Materials

The following supporting information can be downloaded at: https://www.mdpi.com/article/10.3390/toxics12020155/s1, Figure S1: Characterization of polystyrene microplastic. TEM images (A), size distribution (B), FTIR spectra (C), Hydrodynamic diameter, and zeta potential in deionized water (D); Figure S2: Scanning electron microscopy (SEM) images of roots in soybean (A–C) exposed to 50 mg L^−1^ PS-NPs for 7 d and the control of mung bean (D–F). Note: (A) and (D) are partial enlargements of (B) and (E), respectively; (B) and (E) are partial enlargements of (C) and (F), respectively.
