# Supplementary material for: Discrepancy of Growth Toxicity of Polystyrene Nanoplastics on Soybean (Glycine max) and Mung Bean (Vigna radiata)
Source: Toxics. 2024 Feb 17;12(2):155. doi: 10.3390/toxics12020155 (PMC10892715; doi:10.3390/toxics12020155)
Supplement: Supplementary file 1 [file toxics-12-00155-s001.zip › toxics-2817806-supplementary.pdf]

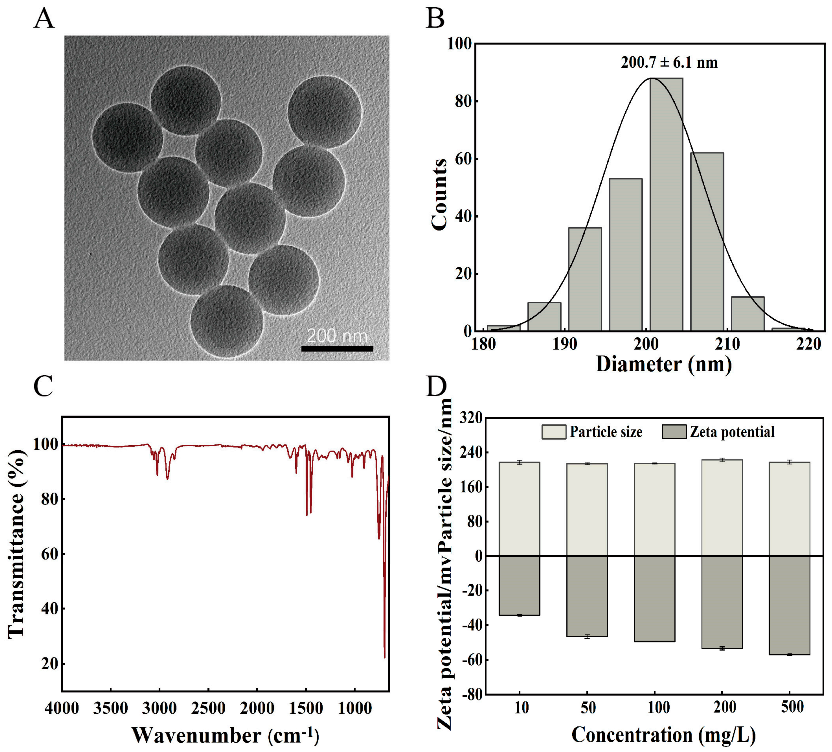

Figure S1. Characterization of polystyrene microplastic. TEM images (A), size distribution (B), FTIR spectra (C), Hydrodynamic diameter, and zeta potential in deionized water (D).

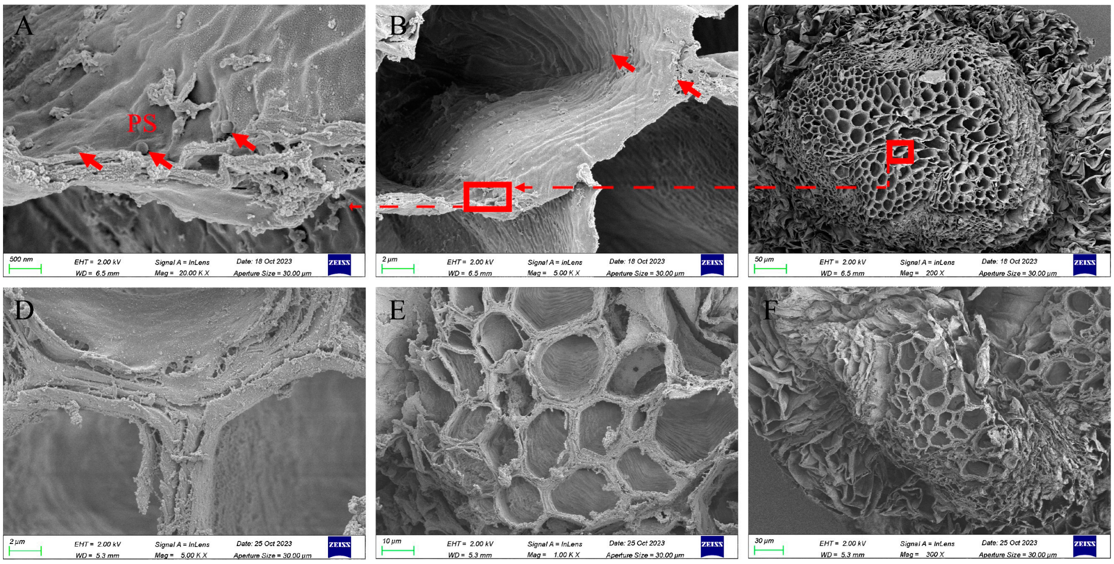

Figure S2. Scanning electron microscopy (SEM) images of roots in soybean (A-C) exposed to 50 mg L^{-1} polystyrene(PS) nanoplastics for 7 d and the control of mung bean (D-F). Note: (A) and (D) are partial enlargements of (B), and (E) respectively; (B), and (E) are partial enlargements of (C), and (F) respectively.
